# Sex-Driven Variation in Polar Metabolites and Lipid Motifs of *Paracentrotus lividus* Gonads Profiled by ^1^H NMR

**DOI:** 10.3390/metabo16030211

**Published:** 2026-03-21

**Authors:** Ricardo Ibanco-Cañete, Estela Carbonell-Garzón, Sergio Amorós-Trujillo, Pablo Sanchez-Jerez, Frutos Carlos Marhuenda Egea

**Affiliations:** 1 Department of Marine Sciences and Applied Biology, University of Alicante, Carretera San Vicente del Raspeig s/n, 03690 Alicante, Spainestela.carbonell@ua.es (E.C.-G.); pablo.sanchez@ua.es (P.S.-J.); 2 Department of Biochemistry and Molecular Biology and Agricultural Chemistry and Edafology, University of Alicante, Carretera San Vicente del Raspeig s/n, 03690 Alicante, Spain

**Keywords:** ^1^H NMR, metabolomics, lipidomics, *Paracentrotus lividus*, sea urchin gonads

## Abstract

**Highlights:**

**What are the main findings?**

- *Paracentrotus lividus* gonads show clear sex-linked ^1^H NMR fingerprints when polar and apolar fractions are profiled together.
- A curated polar metabolite annotation is provided (37 metabolites; 71 assigned resonances), supported by 1D NMR and HSQC confirmation.

**What are the implications of the main findings?**

- The curated peak list and reporting workflow offer a reusable reference framework for reproducible NMR metabolomics in sea urchin gonads and related matrices.
- Sex-linked markers and lipid-motif readouts can be cited as a baseline for comparative studies (season, diet, site, aquaculture conditions) and for future authentication/quality investigations.

**Abstract:**

Background/Objectives: Sea urchin gonads (“roe”) are a valuable seafood product and a chemically complex matrix whose composition varies with physiology and environment. We present a biphasic extraction and ^1^H NMR workflow to build a reusable reference inventory of polar metabolites and apolar lipid features in Paracentrotus lividus. Methods: Gonads from 37 adults (23 males, 14 females) collected at two sites (Alicante and Jávea–Dénia, Spain; October 2024) were lyophilized, extracted with methanol/chloroform/water, and analyzed by 400 MHz ^1^H NMR in buffered aqueous solution (polar) and CDCl_3_ (apolar). Polar metabolite identification combined 1D patterns with database matching and ^1^H–^13^C HSQC confirmation on representative samples, yielding 71 annotated resonances corresponding to 37 metabolites spanning amino acids, osmolytes/quaternary amines, carbohydrates/aminosugars, and nucleoside/purine-related compounds. Results: Polar fingerprints enabled supervised modelling: PLS-LDA separated sexes with low cross-validated error, and SPA/COSS ranking highlighted glycine, alanine, creatine and osmolyte-associated signals as key discriminants; pathway mapping supported the enrichment of amino-acid and one-carbon/purine networks. Apolar spectra were annotated at the motif level and used for lipid-index estimation, indicating substantial unsaturation but low docosahexaenoic acid (DHA) and modest sex effects. Conclusions: The curated peak lists and reporting framework facilitate reproducible NMR annotation and future comparative studies of *P. lividus* gonads.

## 1. Introduction

Sea urchin gonads (“roe”, or “uni”) are a high-value seafood product and are increasingly studied as a reservoir of nutritionally and biologically relevant small molecules, spanning free amino acids and nucleotide-related compounds (linked to taste and freshness), osmolytes, and complex lipids. In the edible Mediterranean species *Paracentrotus lividus*, gonadal biochemical composition is not static: it varies with key biological and environmental drivers, including sex, season and gametogenic stage, with direct implications for quality traits and valorization. Accordingly, gonads constitute a chemically information-rich matrix for integrative characterization and for downstream studies in physiology, quality control and resource management [1,2,3].

From an analytical perspective, metabolomics provides an efficient route to describe the small-molecule composition of biological samples in a comprehensive yet information-dense manner. Among the main platforms, nuclear magnetic resonance (NMR) spectroscopy is particularly attractive because it is inherently quantitative, highly reproducible, and typically requires limited sample manipulation; in addition, structural information is encoded in chemical shifts, J-couplings, and signal multiplicities, which supports both profiling and metabolite identification. However, the same features that make ^1^H NMR efficient for complex mixtures—especially its narrow chemical-shift dispersion—also create practical challenges: extensive peak overlap and matrix-dependent effects can lead to ambiguous assignments if identification relies only on 1D spectra or incomplete reference matching [4]. For this reason, community guidance on metabolomics minimum reporting standards and NMR best practices emphasize transparent documentation of sample preparation and key acquisition/processing parameters, together with explicit, evidence-graded metabolite identification (e.g., 2D confirmation and/or spiking where needed), to ensure reproducibility and cross-study comparability [5,6].

Recent work shows that sea urchin gonadal metabolomes can capture coherent physiological strategies rather than isolated biomarkers. For example, ^1^H HR-MAS NMR applied to intact gonads of two sympatric Mediterranean Sea urchins (*Arbacia lixula* and *Paracentrotus lividus*) revealed systematic, species-linked contrasts consistent with different allocations between osmolyte/redox pools and amino-acid-enriched anabolic reserves [7]. In parallel, complementary “omics” and food-chemistry studies continue to expand the biochemical landscape of edible gonads; in particular, LC–MS lipidomics can now resolve hundreds to thousands of lipid molecular species and identify sex- and quality-related lipid patterns in sea urchins [8,9]. Together, these advances reinforce both the biological relevance and chemical complexity of gonadal tissue—and they also underline a practical bottleneck for NMR-based studies: the need for carefully curated, clearly evidenced peak assignments that enable confident identification (or explicit uncertainty) for each reported feature.

To strengthen metabolite annotation in NMR metabolomics, reference databases such as the Human Metabolome Database (HMDB) and the Biological Magnetic Resonance Data Bank (BMRB) provide curated compound information together with spectral and chemical-shift resources that support candidate generation and sanity checks in complex mixtures. In parallel, open metabolomics repositories such as MetaboLights enable deposition of raw data and rich metadata, facilitating reuse, reanalysis, and transparent benchmarking of annotation strategies across laboratories [10].

Here, we present an integrated ^1^H NMR characterization of *Paracentrotus lividus* gonads using paired polar and apolar extracts to capture both water-soluble metabolites and lipid-dominated spectral features. Adult individuals were collected at two Mediterranean coastal sites (Alicante and Jávea–Dénia, SE Spain) in early October 2024, yielding 37 gonad samples (23 males and 14 females). Building on current metabolomics reporting guidance and NMR best practices [4,5,6], our main aim is to deliver a curated, reusable reference inventory of gonadal features detectable under the described conditions: (i) evidence-graded annotation of polar metabolites across the aliphatic (0.5–5.5 ppm) and aromatic (6.0–9.0 ppm) regions, and (ii) annotation of characteristic apolar lipid motifs (including sterol- and glycerolipid-related signatures), with explicit flagging of overlap-prone segments. Candidate assignments were supported by database resources and 2D-NMR-assisted identification frameworks (HMDB/BMRB and COLMARm-type workflows) [11]. As a biological proof-of-concept for the utility of the curated profiles, we also show that the polar fingerprint contains robust sex-linked signatures, whereas apolar lipid-motif variation is comparatively modest in this dataset. Finally, by aligning the deliverables (annotated peak lists, confidence notes, and metadata) with open-data practices, the study is designed to facilitate transparent reuse and benchmarking in future work on seasonality, diet, environmental stressors, and processing/valorization of *P. lividus* gonads [10].

The main output of this study is a curated, evidence-graded ^1^H NMR peak list for *P. lividus* gonads, reporting chemical shifts and multiplicities for polar metabolites together with a practical annotation of apolar lipid motifs [12,13,14,15,16]. By explicitly documenting overlap-prone regions and assigning confidence levels, the resource is intended to be directly citable and reusable as a reference for peak interpretation and cross-study comparison in sea urchin gonad metabolomics [4,6].

Beyond annotation, the curated framework enables hypothesis-driven reuse: it provides a baseline to test how gonad chemistry shifts with seasonality, gametogenic stage, diet, habitat quality, and environmental stressors, and it supports transparent benchmarking of annotation strategies across laboratories. In line with open metabolomics practice, the reporting structure is designed to facilitate data and metadata deposition and subsequent reanalysis in public repositories.

## 2. Materials and Methods

### 2.1. Biological Material and Study Design

Adult *Paracentrotus lividus* specimens were collected by snorkel diving at 1–6 m depth along the Mediterranean coast of Alicante Province (Alicante and Jávea-Denia, SE Spain) during the same sampling week in October 2024 (2 October 2024 in Jávea-Denia and 8 October 2024 in Alicante). Two habitat types (natural vs. artificial) were initially considered at each locality, although habitat/location stratification was not pursued further due to the lack of consistent effects in downstream analyses. Individuals (>40 mm test diameter) were transported alive to the laboratory in portable coolers containing aerated seawater in order to preserve field temperature conditions as much as possible during transport. Upon arrival, specimens were euthanized by freezing, biometrically recorded (size and wet weight), sexed after dissection, and gonads were excised and stored frozen until processing. The inclusion of more than one coastal sector was intended to reduce spatial pseudoreplication and to avoid over-representing a single local cove or collection point as a proxy for the wider Alicante coastal area. Sampling was therefore distributed across localities selected to represent broadly comparable shallow Mediterranean conditions. Natural habitats consisted of shallow rocky areas with algal growth, whereas artificial habitats corresponded to harbour breakwaters formed by large concrete blocks. The overall design was approximately balanced between natural and artificial habitats. Although locality and habitat category were considered during exploratory data analysis, PCA did not reveal any clear or consistent clustering associated with either factor; accordingly, these variables were not retained as stratifying factors in the final chemometric models, and all individuals were treated as a single cohort for the purposes of the present study.

Gonadal tissues used in this study originated from the same specimen collection framework previously employed for gonad profiling in *P. lividus* [17]. A total of 37 gonad samples were analyzed (23 males, 14 females). The biological and biometric characteristics of the cohort, including test diameter, test height, total wet weight, gonad weight, and gonadosomatic index (GSI), were recorded and summarized in Table 1.

All procedures involving *Paracentrotus lividus* specimens were conducted within the framework of the research project under which sampling and analysis were authorized. Sea urchins were collected, transported, handled, and dissected in accordance with institutional and applicable ethical guidelines for marine invertebrate research. The study protocol was reviewed and approved by the Ethics Committee of the University of Alicante (UA-2024-10-10).

### 2.2. Lyophilization and Sample Preparation

Frozen gonadal tissues were lyophilized for 24 h. Lyophilized material was homogenized to a fine powder. For each specimen, 50 mg of powdered gonad was transferred into a 1.5 mL microcentrifuge tube for biphasic extraction.

### 2.3. Biphasic Extraction of Polar and Apolar Metabolites

Polar and apolar metabolites were extracted using a methanol/chloroform/water biphasic protocol [18]. Briefly, 400 µL of methanol and 85 µL of water were added to each tube (note: methanol was used; NaOH was not used in this step). Samples were vortex-mixed for 60 s at maximum speed and sonicated for 10 min. Then, 200 µL chloroform was added, followed by vortex mixing (60 s). Samples were incubated on ice for 5 min and sonicated again for 10 min. Subsequently, an additional 200 µL chloroform and 200 µL water were added, vortexed (60 s), and sonicated (10 min). Phase separation was achieved by centrifugation at 12,000× *g* for 10 min at 4 °C, yielding an upper polar (aqueous) phase and a lower apolar (organic) phase.

The lower apolar phase was collected using disposable syringes and transferred into labelled tubes. The upper polar phase was transferred using a micropipette into labelled tubes. Both fractions were dried to completeness in a SpeedVac benchtop centrifuge (Eppendorff, Hamburg, Germany) (apolar fraction at ~30 °C with open caps; polar fraction to complete removal of aqueous solvent).

### 2.4. NMR Sample Preparation

For the apolar fraction, dried extracts were reconstituted in 500 µL deuterated chloroform (CDCl_3_) and transferred into 5 mm NMR tubes, sealed to avoid solvent evaporation.

For the polar fraction, dried extracts were reconstituted in 600 µL phosphate buffer (50 mM) containing sodium azide, and 60 µL D_2_O was added for field locking. After a brief centrifugation (3 min at 12,000 rpm), supernatants were transferred to 5 mm NMR tubes and sealed.

### 2.5. ^1^H NMR Data Acquisition

All ^1^H NMR experiments were performed on a Bruker Avance 400 MHz spectrometer equipped with a 5 mm HBB^13^C TBI probe and actively shielded z-gradients (Bruker, Rheinstetten, Germany). One-dimensional ^1^H spectra were acquired at 298 K using a recycle delay of 2 s, 32,768 time-domain points, an acquisition time of 2.556 s, and 64 scans per sample. Spectra were apodized with an exponential line broadening of 0.3 Hz prior to Fourier transformation. For polar extracts, spectra were referenced to TSP (0.5 mM) at 0.00 ppm. For apolar extracts in CDCl_3_, spectra were referenced to the residual solvent signal (as applicable for chloroform-based NMR).

### 2.6. Spectral Processing and Data Handling

Raw NMR data were processed in TopSpin 4.3.0 (Bruker, Rheinstetten, Germany) and exported as text files. Spectra were adjusted to a consistent chemical shift window (11.0 to −0.5 ppm) prior to multivariate handling. Processed datasets were imported into MATLAB version 2024 (MathWorks, Natick, MA, USA) for downstream preprocessing and statistical analysis.

### 2.7. Metabolite Annotation Strategy

Metabolite assignments were performed primarily from ^1^H chemical shift patterns, multiplicities, and coupling information, supported by public reference databases (e.g., HMDB (https://hmdb.ca/, accessed on 18 February 2026)) and BMRB (http://bmrb.io/, accessed on 18 February 2026)) and relevant literature comparisons. When peak overlap or ambiguity prevented confident assignment from 1D ^1^H spectra alone, signals were conservatively left unassigned (and flagged for future confirmation by 2D NMR if required).

### 2.8. Statistical Analysis and Chemometric Modelling

Processed ^1^H NMR datasets (polar and apolar fractions) were handled in MATLAB (MathWorks, Natick, MA, USA). For multivariate analysis of the polar and apolar fraction, the input matrix was constructed from the processed 1D spectra in the frequency domain, exported from TopSpin 4.3.0 (Bruker, Rheinstetten, Germany) after Fourier transformation, phase/baseline correction, referencing, and spectral windowing. Accordingly, each sample was represented by its spectral intensity values across a common chemical-shift axis, yielding a full-resolution pointwise spectral matrix rather than a conventional coarse-binned dataset. Prior to modelling, spectra were inspected for artefacts and, when appropriate, narrow regions dominated by non-informative signals (e.g., residual solvent/standard peaks) were excluded to avoid undue leverage on supervised models. Data were mean-centred and scaling was evaluated as part of model validation (Pareto scaling and autoscaling, as specified in the corresponding validation outputs).

Supervised classification was performed using Partial Least Squares–Linear Discriminant Analysis (PLS-LDA) implemented with the libPLS workflow in MATLAB (https://www.libpls.net/, accessed on 18 February 2026). In this framework, PLS latent variables (LVs) were computed using the NIPALS algorithm and class separation was obtained by applying linear discriminant analysis (LDA) to the latent-variable scores. Model dimensionality (optimal number of LVs) and predictive performance were assessed using internal validation routines, including cross-validation and Monte Carlo cross-validation (MCCV). Model quality was summarized using standard classification metrics (misclassification error, sensitivity, specificity) and ROC-derived AUC values [19].

To move from full-spectrum discrimination to an interpretable set of candidate markers, a Subwindow Permutation Analysis (SPA) was applied to the peak-level dataset derived from the curated polar resonance table (Table 2). SPA is a supervised Monte Carlo variable-selection approach that repeatedly builds PLS-LDA sub-models on randomly sampled subsets of samples and variables, and evaluates the statistical contribution of each variable by comparing prediction errors between the original and permuted datasets. For each variable, SPA provides a nominal *p*-value and the corresponding Conditional Synergistic Score (COSS = −log_10_ p). Unless otherwise stated, variables meeting the nominal criterion *p* < 0.05 were retained as statistically supported contributors for downstream reporting and visualization [20,21].

For univariate group comparisons (e.g., selected peak intensities/integrals and apolar lipid indices), a two-sided Mann–Whitney U test was used given the non-guaranteed normality of metabolomics readouts. Exact *p*-values are reported. Boxplots (where shown) were generated using the standard convention: centre line = median, box = interquartile range (IQR), whiskers = 1.5 × IQR, and individual points represent sample-level values.

### 2.9. Pathway Analysis (MetaboAnalyst)

Pathway analysis was carried out using MetaboAnalyst v. 6.0 (Pathway Analysis module; www.metaboanalyst.ca, accessed on 18 February 2026) in “compound list” mode with the KEGG pathway library. Metabolite names were mapped to KEGG identifiers using the built-in name matching tools [22,23]. Enrichment was assessed by over-representation analysis (hypergeometric test), and pathway topology was evaluated using relative betweenness centrality to compute pathway impact scores.

Importantly, the input compound list comprised all metabolites identified in the ^1^H NMR spectra of the polar extracts across the complete dataset (i.e., considering both males and females), in order to summarize the biochemical landscape represented in the polar gonadal metabolome under the present analytical conditions. For each pathway, MetaboAnalyst outputs (where available) included total compounds, expected hits, observed hits, raw *p*-value, −log_10_(p), multiple-testing adjusted *p*-values (Holm) and FDR, together with pathway impact. Complete pathway statistics are reported in the Supplementary Materials table associated with the MetaboAnalyst output.

### 2.10. Apolar Extract Quantification and Lipid Index Calculation

For the apolar fraction (CDCl_3_), lipid-class indices were computed from integrals of diagnostic spectral regions using the balance-equation scheme described by Bratu et al. [24]. Briefly, integrals were obtained by numerical integration of predefined chemical shift windows corresponding to characteristic lipid motifs (e.g., terminal methyls, methylene envelope, allylic/bis-allylic methylenes, olefinic protons, and DHA-associated sub-regions). In this work, a narrower F′ window (2.37–2.40 ppm) was used to better isolate DHA-related contributions. The resulting system of equations yields molar estimates for total unsaturated and saturated fractions, ω-3 content, and DHA, with internal constraints enabling automated flagging of spectra that violate expected bounds. Spectra failing internal consistency checks were excluded from downstream comparisons.

Sex-stratified comparisons of the derived apolar indices were performed using two-sided Mann–Whitney testing. When multivariate separability of the apolar indices was assessed, classification performance was evaluated under cross-validation to avoid optimistic estimates.

## 3. Results

### 3.1. ^1^H NMR Spectral Overview and Metabolite Annotation (Polar Extracts)

**Figure 1:**
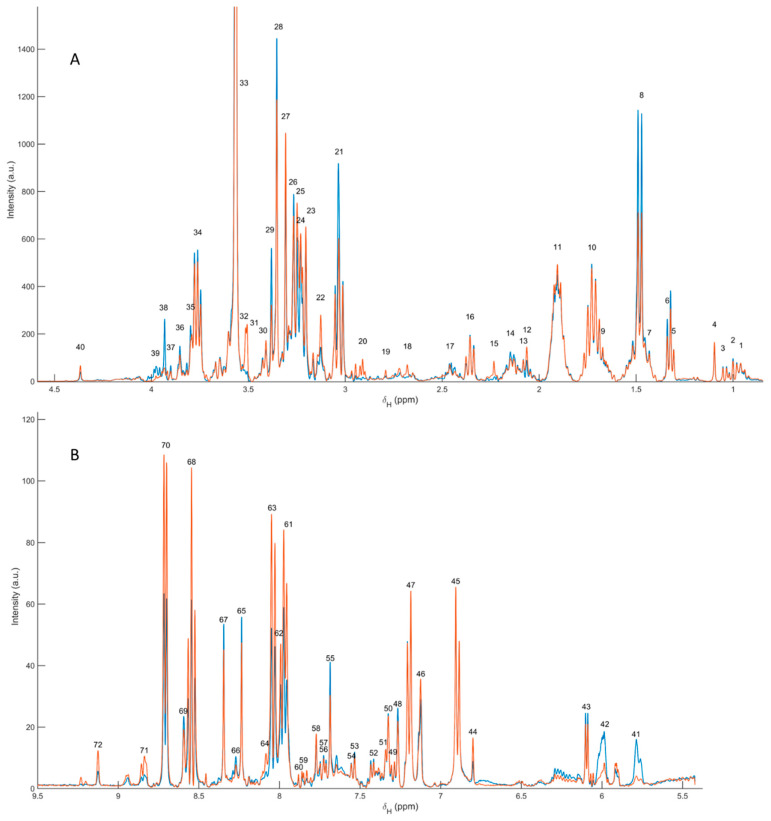
Representative ^1^H NMR spectra of polar extracts from *Paracentrotus lividus* gonads (female (orange line) and male (blue line) examples), acquired at 400 MHz and 298 K in phosphate buffer and referenced to TSP (0.00 ppm). (**A**) Aliphatic and carbohydrate-rich region, showing the main annotated amino acids, osmolytes, amines, and carbohydrate-related resonances (0.5–5.5 ppm) and (**B**) aromatic/downfield region, showing annotated aromatic, nucleoside-, purine-, pyrimidine-, and heteroaromatic resonances (6.0–9.0 ppm) regions. Female and male representative spectra are shown in orange and blue, respectively. Peak identities are listed in Table 2.

Representative ^1^H NMR spectra of the polar extracts obtained from *Paracentrotus lividus* gonads (males and females) showed a consistent metabolomic fingerprint dominated by low-molecular-weight metabolites, with the expected distribution of resonances across the aliphatic (≈0.8–3.0 ppm), carbohydrate/heteroatom-rich (≈3.0–5.5 ppm) and aromatic (≈6.0–9.0 ppm) regions. Metabolite assignment was supported by 2D NMR experiments (including ^1^H–^13^C HSQC on representative samples) and by comparison with reference databases and literature patterns. The complete list of assigned resonances, chemical shifts, multiplicities and moiety-level annotations is provided in Table 2, while a summary figure with labelled signals is included as Figure 1 (polar fraction).

Using the curated peak table, a total of 71 resonances were annotated, corresponding to 37 unique polar metabolites (64 peaks assigned; remaining peaks left as unassigned due to overlap or insufficient 2D support). The identified metabolite space covered major biochemical categories relevant to gonadal physiology, including: (i) free amino acids and nitrogen carriers (e.g., alanine, valine/isoleucine, threonine, arginine, lysine, glutamate, glutamine, glycine, histidine, phenylalanine, tyrosine and tryptophan); (ii) osmolytes and quaternary amines (taurine, betaine, choline, trimethylamine and trimethylamine N-oxide); (iii) energy-buffer and related metabolites (creatine); (iv) carbohydrates and aminosugars (trehalose, N-acetylglucosamine); (v) nucleotide-related metabolites (uridine/uracil, inosine, hypoxanthine, xanthine, adenosine monophosphate and 3,7-dimethyluric acid); and (vi) aromatic and diet-/microbiota-linked compounds (e.g., trigonelline, kynurenine, vanillic acid), together with small one-carbon/methylated species detected in the low-ppm region (methanol, methylamine).

Overall, these assignments establish a chemically coherent polar metabolite panel suitable for (a) untargeted multivariate modelling using the full ^1^H NMR spectral profiles, and (b) targeted/semi-targeted analyses based on peak heights or integrations of the annotated metabolites (Section 3.2, Section 3.3 and Section 3.4).

### 3.2. PLS-LDA Model

**Figure 2:**
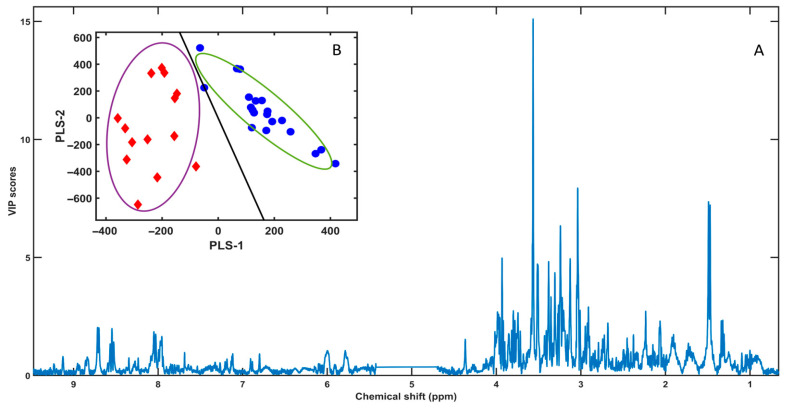
(**A**) VIP profile obtained from the PLS-LDA of polar-extract ^1^H NMR spectra from male and female *Paracentrotus lividus* gonads. Peaks/regions with the highest VIP values indicate the spectral variables with the strongest discriminatory power between sexes and were used to guide downstream SPA-based ranking. (**B**) PLS-LDA score plot (LV1 vs. LV2) showing sample clustering by sex, with female samples represented as red diamonds and male samples as blue circles. The continuous line corresponds to the linear discriminant boundary of the PLS-LDA model; ellipses indicate the 95% confidence regions for each group.

To assess whether male and female *Paracentrotus lividus* gonads can be discriminated based on the polar-fraction ^1^H NMR fingerprints, a Partial Least Squares–Linear Discriminant Analysis (PLS-LDA) model was built using the full spectral matrix in the frequency domain (Figure 2), i.e., the retained pointwise intensity variables across the common chemical-shift window after exclusion of narrow non-informative regions. The PLS-LDA implementation followed the libPLS framework, in which PLS scores are obtained by the NIPALS algorithm after data pretreatment, and class separation is achieved by applying linear discriminant analysis to the latent variables; model outputs include VIP scores and ROC-derived metrics (AUC, sensitivity, specificity) [19].

An exploratory fit computed up to six latent variables returned R^2^X = [0.1837, 0.3028, 0.0641, 0.0515, 0.0423, 0.0427] and R^2^Y = [0.7525, 0.0479, 0.0830, 0.0504, 0.0189, 0.0075], showing that class-related variance was captured predominantly by LV1 (R^2^Y = 0.7525). When focusing on the dimensionality supported by validation, the first two latent variables already accounted for R^2^X (cum) = 0.4865 and R^2^Y (cum) = 0.8004, indicating that a compact latent space was sufficient to encode most sex-associated information.

The PLS score plot (PLS-1 vs. PLS-2) showed a clear separation between groups, with female samples (red diamonds) clustering at negative PLS-1 values and male samples (blue circles) at positive PLS-1 values (Figure 2B). Apparent (training) performance for the fitted model yielded error = 0, sensitivity = 1, specificity = 1 and AUC = 1. However, to provide a more conservative estimate of predictive performance, cross-validation (CV) and Monte Carlo cross-validation (MCCV) were carried out. Under CV (pareto scaling), the optimal model complexity was optLV = 2, achieving error_min = 0.0294 (≈2.94% misclassification across *n* = 34 samples), with sensitivity = 1, specificity = 0.9375, and AUC = 1. Under MCCV (autoscaling), the optimal complexity was again optLV = 2, with error_min = 0.0793. Overall, both validation approaches supported that the discrimination between sexes is robust and is achieved with a low number of latent variables, mitigating overfitting concerns while preserving separation.

The corresponding VIP profile (Figure 2A) highlighted the spectral regions contributing most strongly to discrimination, with the largest VIP features concentrated in the 3.0–4.2 ppm range (carbohydrate/heteroatom-rich region), alongside additional contributions from aliphatic and aromatic resonances. These VIP-enriched regions guided the subsequent targeted variable selection and biomarker ranking by Subwindow Permutation Analysis (SPA) and COSS (Section 3.3 and Section 3.4) [19,20,21].

### 3.3. SPA-Based Variable Selection and COSS Ranking

To move from untargeted discrimination (full-spectrum PLS-LDA) to a more interpretable set of sex-discriminant markers, Subwindow Permutation Analysis (SPA) was applied to the peak-level dataset derived from the curated polar ^1^H NMR table (Table 2). SPA evaluates the statistical contribution of each variable to the predictive performance of the classification model by iteratively permuting local subwindows, yielding a variable-wise *p*-value and the associated Conditional Synergistic Score (COSS), commonly defined as COSS = −log10(p) [21].

**Figure 3:**
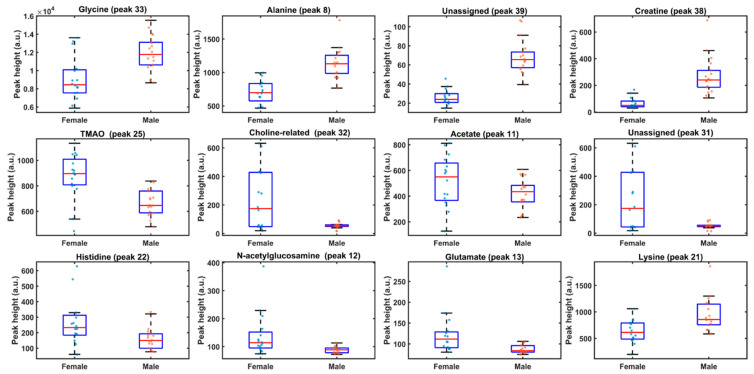
Sex-stratified boxplots of selected polar ^1^H NMRs (peak heights) in *Paracentrotus lividus* gonads. Peak heights (a.u.) were extracted at the indicated chemical shifts (ppm) from the polar ^1^H NMR spectra and are shown for females (*n* = 14) and males (*n* = 23). Panels correspond to representative discriminant resonances/assignments (Table 2): glycine (3.5648 ppm), alanine (1.4715 ppm), creatine (3.9323 ppm), TMAO (3.2488 ppm), a choline-related signal (3.5101 ppm), acetate (1.9052 ppm), histidine (3.1273 ppm), N-acetylglucosamine (2.0630 ppm), glutamate (2.0816 ppm), lysine (3.0362 ppm), and two unassigned features (3.9800 and 3.5042 ppm). Boxes represent the interquartile range (IQR) with the median as the centre line; whiskers extend to 1.5 × IQR; points denote individual samples.

Based on the SPA output, a total of 14 variables met the nominal significance criterion *p* < 0.05 (corresponding to COSS > 1.30) and were retained as the most informative spectral features for sex discrimination (Figure 3). The highest-ranked marker was the glycine resonance at δH = 3.5648 ppm (*p* = 7.41 × 10^−18^; COSS = 17.13), followed by alanine at δH = 1.4715 ppm (*p* = 3.16 × 10^−14^; COSS = 13.50) and creatine at δH = 3.9323 ppm (*p* = 2.32 × 10^−9^; COSS = 8.63). Additional significant variables clustered predominantly in the 3.0–3.6 ppm region, consistent with the VIP-enriched zone observed in the full-spectrum PLS-LDA model, and included signals assigned to histidine (δH = 3.1273 ppm), lysine (δH = 3.0362 ppm; plus a lysine-associated aliphatic signal at 1.9052 ppm), and multiple osmolyte/quaternary-amine-related resonances such as betaine (δH = 3.2298 ppm), TMAO (tentative) (δH = 3.2488 ppm), and a choline-related feature (δH = 3.5101 ppm). A statistically supported contribution from the aromatic region was also detected for purine riboside/ribonucleotide-like resonances (δH = 8.2351 ppm), pointing to nucleotide-related metabolism as part of the discriminatory signature.

Notably, several high-ranking SPA variables corresponded to currently unassigned or partially overlapped resonances (e.g., δH ≈ 3.5042 and 3.9800 ppm), indicating that part of the discriminant information resides in crowded regions where attribution remains uncertain; these features were nonetheless kept in the ranked list to preserve predictive signal and were treated cautiously in biological interpretation. Overall, SPA/COSS ranking corroborated the main VIP-driven spectral zones and provided a statistically grounded short-list of candidate biomarkers for downstream visualization and group-wise comparison (Section 3.4).

### 3.4. MetaboAnalyst Pathway Representation of the Annotated Polar Metabolome

**Figure 4:**
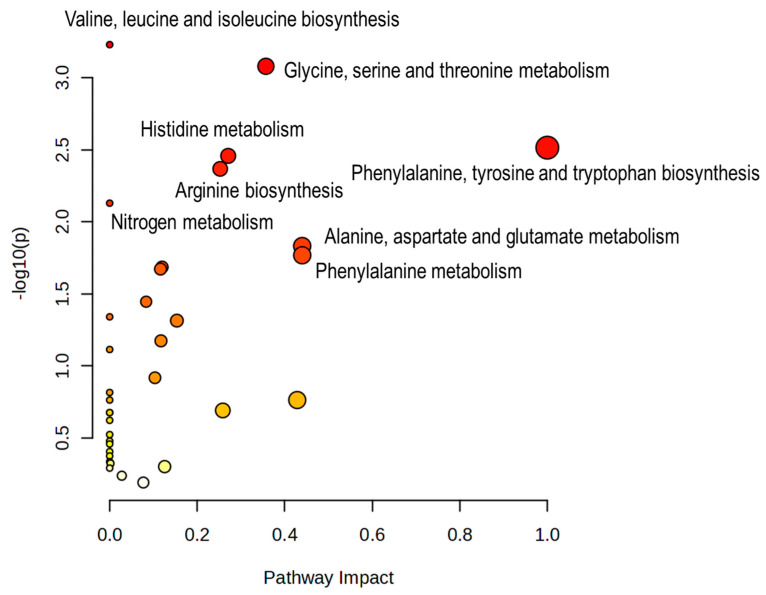
Pathway analysis of the annotated polar metabolite set using MetaboAnalyst. Bubble plot generated from the complete list of annotated polar metabolites detected in the ^1^H NMR spectra of *Paracentrotus lividus* gonads across the full cohort. The x-axis shows pathway impact derived from topology analysis, and the y-axis shows pathway significance expressed as −log10 (raw *p*-value). Bubble size reflects the number of matched metabolites within each pathway, and colour indicates enrichment significance. Because the input consisted of the full annotated polar metabolite inventory rather than a subset of sex-discriminatory features, the plot should be interpreted as a representation of pathway coverage under the present analytical conditions (Supplemetary Table S1).

To contextualize the biochemical landscape captured in the polar gonadal extracts, the complete set of annotated polar metabolites was analyzed using the MetaboAnalyst Pathway Analysis module (KEGG library; compound-list mode), combining over-representation analysis with pathway-topology metrics. The resulting bubble plot (Figure 4) summarizes pathway significance (−log10 of the raw *p*-value) versus pathway impact, where bubble size reflects the number of matched metabolites and colour encodes the enrichment *p*-value. Because the input list comprised all annotated polar metabolites detected across the full cohort, the results should be interpreted as a representation of pathway coverage under the present analytical conditions rather than as direct evidence of sex-specific pathway directionality.

The most represented pathways were valine, leucine and isoleucine biosynthesis (3/8 matched metabolites; raw *p* = 5.30 × 10^−4^; FDR = 0.0403), phenylalanine, tyrosine and tryptophan biosynthesis (2/4; raw *p* = 0.00285; impact = 1.00), histidine metabolism (3/14; raw *p* = 0.00315; impact = 0.271), purine metabolism (6/71; raw *p* = 0.00385; impact = 0.237), arginine biosynthesis (3/15; raw *p* = 0.00387; impact = 0.253), and glycine, serine and threonine metabolism (4/34; raw *p* = 0.00597). Additional pathway representation was observed for nitrogen metabolism (2/6; raw *p* = 0.00692) and alanine, aspartate and glutamate metabolism (3/23; raw *p* = 0.0134; impact = 0.440). Overall, the pathway profile was dominated by amino-acid and nitrogen-related metabolism, with additional contributions from purine/nucleotide-linked chemistry.

These results provide a biochemical context for the annotated polar metabolome detected by ^1^H NMR, but they should not be overinterpreted as pathway-level proof of sex-driven regulation, since the input to MetaboAnalyst was not restricted to sex-discriminatory metabolites.

### 3.5. ^1^H NMR Spectral Overview and Metabolite Annotation (Apolar Extracts)

The apolar extracts recorded in CDCl_3_ showed spectra dominated by lipid resonances, consistent with a mixture of neutral lipids (primarily triacylglycerols, TAG, and diacylglycerols, DAG), fatty acyl chains (FA), and membrane-associated glycerophospholipids (PL). The aliphatic region (≈0.6–2.3 ppm) was characterized by intense terminal methyl signals (≈0.88–0.98 ppm) and the methylene envelope of long acyl chains (≈1.26–1.32 ppm), together with β-methylene contributions (≈1.60–1.70 ppm) and allylic methylenes (≈2.02–2.08 ppm) indicative of unsaturated fatty acids. The α-methylene to the carbonyl group appeared around ≈2.31 ppm, with a distinct narrow sub-region at ≈2.37–2.40 ppm used as the diagnostic F′ window for DHA-related acyl contributions in the Bratu scheme. Bis-allylic methylenes of polyunsaturated chains were observed at ≈2.77–2.84 ppm, while the olefinic region around ≈5.28–5.37 ppm reflected the overall unsaturation and included contributions from TAG glycerol (sn-2 CH) and –CH=CH– protons.

**Figure 5:**
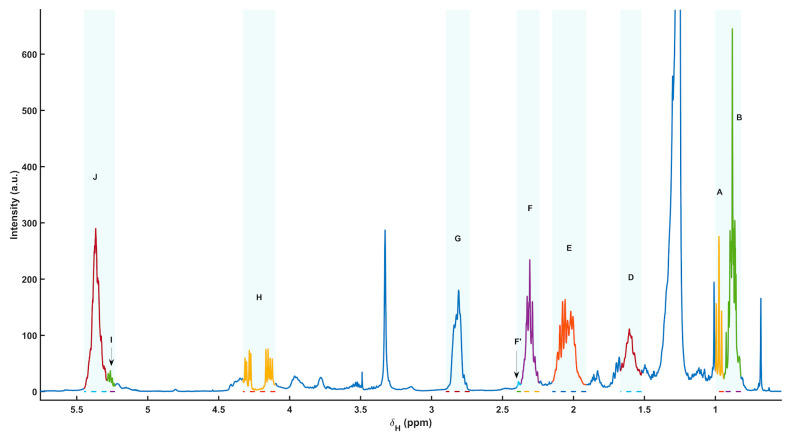
Representative ^1^H NMR spectrum of apolar extracts recorded in CDCl_3_, dominated by lipid resonances. The lettered regions (A–J and F′) follow the integration scheme of Bratu et al. [24] and are shown as coloured bands to indicate the spectral windows numerically integrated for lipid-index calculations. In this scheme, A corresponds to ω-3 terminal methyl protons, B to non-ω-3 terminal methyls, C to the bulk acyl-chain methylene envelope, D to β-methylene protons adjacent to carbonyl groups, E to allylic methylenes, F to α-methylene protons adjacent to carbonyl groups, F′ to the DHA-sensitive sub-region, G to bis-allylic methylenes of polyunsaturated fatty acids, H to the glycerol CH_2_–O–CO region, I to the glycerol sn-2 CH–O–CO region, and J to olefinic –CH=CH– protons. Thus, the coloured regions mark the exact portions of the spectrum integrated for each Bratu-type variable [24]. Annotated motifs include terminal methyls, acyl-chain methylene envelope, allylic and bis-allylic methylenes, olefinic protons, TAG/DAG glycerol –CH_2_–O–CO signals, and phospholipid-associated glycerophospholipid fragments. Moiety-to-lipid-class mapping is summarized in Table 3.

In the heteroatom-rich region (≈3.5–4.4 ppm), several resonances supported the presence of glycerol backbones and phospholipid moieties. Signals in the ≈4.10–4.33 ppm interval correspond to CH_2_–O–CO motifs typical of TAG/DAG, whereas broader features at ≈3.78–3.95 ppm and near ≈4.38 ppm are consistent with phospholipid-associated –CH_2_–O–P– and related glycerophospholipid fragments, as reported in recent lipid NMR annotation frameworks (Figure 5) [13,14,15,16]. Overall peak assignments, chemical shifts (corrected), and moiety-to-lipid-class mapping are summarized in Table 3, providing a coherent annotation layer for subsequent equation-based lipid class estimations [24].

### 3.6. Lipid Class Estimation Using Bratu Equations and Comparison with Fish Oils

To enable a direct comparison with published fish-oil datasets, lipid class metrics were calculated using the set of nine balance equations described by Bratu et al., based on integrals of diagnostic windows (Figure 5, apolar integration map), including the narrower F′ region (2.37–2.40 ppm) adopted here to better isolate DHA-associated contributions in our spectra. The method yields molar percentages for total unsaturated (n), saturated (s), ω-3 fatty acids (ω_3_), and DHA (h), together with internal constraints (e.g., mass-balance relationships) that allow automated flagging of spectra violating the expected bounds. After excluding three spectra flagged as out-of-range, sea urchin gonad apolar extracts displayed a median unsaturated fraction of 61.6 mol% (IQR 55.8–71.6) and a complementary saturated fraction of 38.4 mol% (IQR 28.4–44.2). The ω-3 fraction reached a median of 20.7 mol% (IQR 18.5–24.0), while DHA showed consistently low levels with a median of 1.71 mol% (IQR 1.40–1.93; range 0.91–2.83) [24].

**Figure 6:**
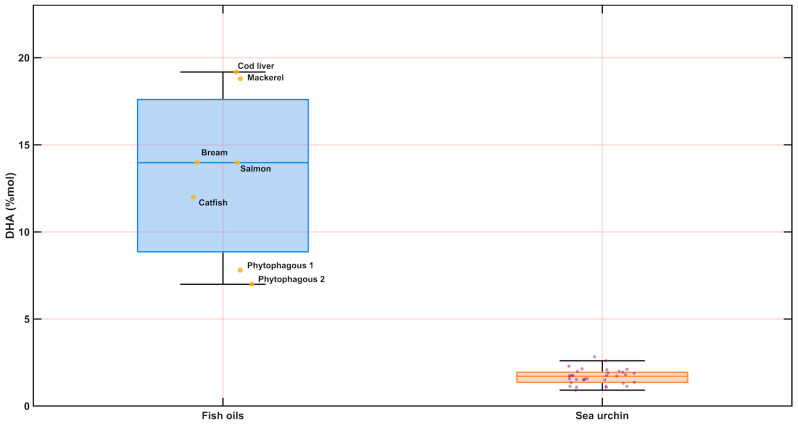
Benchmarking of Bratu-derived DHA (mol%) in apolar extracts of *Paracentrotus lividus* gonads against published fish-oil reference values. DHA (mol%) was estimated from apolar ^1^H NMR spectra (CDCl_3_) by integrating diagnostic lipid regions and applying Bratu-type balance equations (see Methods; integration windows defined in the apolar integration map). The sea urchin dataset (*n* = 34) is shown as a boxplot with individual sample points; fish oils are displayed as reference values reported in Bratu et al., with labelled examples (e.g., cod liver, mackerel, bream, salmon, catfish; phytophagous oils). Boxes represent the interquartile range (IQR) with the median as the centre line; whiskers extend to 1.5 × IQR; points denote individual observations, highlighting markedly lower DHA in sea urchin gonad apolar extracts relative to fish oils.

When benchmarked against the fish oils reported by Bratu et al. (Table 3 in that study) [24], sea urchin gonad extracts overlapped partially in global unsaturation but diverged strongly in DHA enrichment (Figure 6). In the fish oils, DHA spans approximately 7.0–19.2 mol% (median ~14.0 mol%), i.e., about one order of magnitude higher than in the sea urchin gonads, whereas ω-3 totals in fish oils (≈20.9–35.7 mol%; median ~26.7 mol%) remain systematically above the sea urchin distribution. Collectively, these results indicate that *Paracentrotus lividus* gonad apolar extracts are comparatively depleted in DHA relative to fish oils, despite maintaining substantial overall unsaturation typical of lipid-rich tissues.

### 3.7. Sex-Related Differences and Multivariate Separability (Apolar Metrics)

Sex-stratified comparisons of the Bratu-derived metrics (QC-filtered dataset) indicated broadly similar lipid-class composition between females and males. Mann–Whitney testing showed no significant differences for total unsaturation (Female median 57.9 vs. Male 66.6 mol%; *p* = 0.141), saturation (Female 42.1 vs. Male 33.4 mol%; *p* = 0.141), or DHA (Female 1.52 vs. Male 1.74 mol%; *p* = 0.357). The ω-3 fraction showed a modest shift (Female 19.4 vs. Male 21.6 mol%; *p* = 0.0247), but the effect size is limited and does not translate into robust multivariate separation. Consistently, linear discriminant analysis applied to the apolar feature set yielded only moderate cross-validated discrimination, supporting the conclusion that—within the resolution of these global lipid-class estimates—male and female gonads display largely overlapping apolar lipid profiles.

## 4. Discussion

Overall, the results indicate that the polar gonad metabolome contains robust sex-linked ^1^H NMR signatures, while apolar lipid-motif variation is comparatively modest in this dataset; together with the curated peak list, this establishes a reproducible reference point for future comparative and applied studies on *P. lividus* roe composition.

### 4.1. Multivariate Evidence of Robust Sex Separation and Model Validation

Using the full ^1^H NMR spectral space from polar extracts, the supervised PLS-LDA model achieved a clear discrimination between female and male gonads (Figure 2B), with high explained variance in Y (R2Y dominated by the first latent variable) and an apparent absence of misclassification in the training set. While such perfect or near-perfect class separation can occur when sex is a strong biological driver, it is also a well-known scenario where overfitting may inflate performance if validation is not sufficiently rigorous. For this reason, the cross-validation strategy and diagnostic statistics are critical [25,26]. In this context, the consistently low error rates observed under cross-validation and Monte Carlo cross-validation (MCCV), together with strong sensitivity/specificity and ROC-derived AUC values, show that the model captures a stable sex-associated signal rather than a purely chance configuration of latent variables [25,27]. As a recommended strengthening step (particularly when AUC approaches 1), metabolomics best practice commonly includes label-permutation testing and/or double cross-validation to further bound the risk of overfitting and to document that class structure is not recoverable after class randomization [25,26,27].

### 4.2. Discriminant Variables Converge on Osmolyte Balance, Amino-Acid Pools, and Energy Buffering

A complementary, peak-level strategy (subwindow permutation analysis, SPA, with COSS ranking) prioritized a small set of resonances that best classify sex (Figure 3). This supervised Monte Carlo variable-selection framework is specifically designed to identify variables with reproducible predictive contribution across repeated resampling/permutation cycles, thereby improving interpretability beyond full-spectrum discrimination [20]. Importantly, the SPA/COSS shortlist converges with the overall VIP structure (Figure 2A), indicating that separation is supported by coordinated variation across multiple metabolite families rather than by a single narrow region.

Among the top discriminant peaks, glycine (3.5648 ppm) and alanine (1.4715 ppm) are prominent, alongside creatine (3.9323 ppm) and lysine signatures. In marine invertebrates, free amino acids are frequently major contributors to osmotic balance, nitrogen handling, and tissue remodelling; accordingly, shifts in glycine/alanine pools are consistent with sex-dependent differences in osmolyte budgeting and/or the relative contribution of somatic versus gametic compartments within gonads [28,29]. Conversely, betaine (3.2298 ppm) and TMAO (3.2488 ppm, tentative) also appear among the strongest discriminant signals. These quaternary amines are classical compatible/counteracting osmolytes that stabilize macromolecules and modulate protein–solvent interactions, making them plausible drivers of coherent sex-linked separation in the polar metabolome [29]. The creatine signal is also biologically interpretable in echinoderms, which rely on phosphagen systems (arginine kinase/creatine kinase-type buffering) to match variable energetic demand; differences in phosphagen pools can therefore reflect divergent energy buffering requirements during gametogenesis [30].

### 4.3. Pathway Analysis: Strong Amino-Acid and One-Carbon Signatures, with an Important Caveat on Directionality

When the full set of annotated polar metabolites is queried in MetaboAnalyst pathway analysis (compound-list mode; KEGG library), the over-representation results are dominated by amino-acid metabolism (e.g., glycine/serine/threonine; histidine; alanine/aspartate/glutamate; arginine-related routes), with additional representation of one-carbon-associated and nucleotide-linked pathways (purine/pyrimidine) (Figure 4) [22,23]. This configuration is biologically plausible for gonadal tissue, where metabolic investment in amino-acid trafficking, osmolyte control, and biosynthetic precursors is expected to be substantial across sexes.

However, because the pathway analysis was performed using a combined compound inventory (metabolites detected across both males and females), these results should be interpreted strictly as pathway representation/coverage under the current analytical conditions, not as sex-specific directionality (i.e., not “up-regulated in females” or “up-regulated in males”) [22]. Directionality in the present study is instead provided by the univariate patterns and boxplots for discriminant metabolites (Figure 3). A high-value refinement for the final version would be to run two additional pathway analyses using female-enriched vs. male-enriched metabolite subsets (or ranked lists derived from effect size + significance), enabling a more explicit sex-linked functional narrative while keeping the current “inventory/landscape” analysis as a reference baseline [22].

### 4.4. Integrating Sex Biology of P. lividus Gonads with the Observed Metabolite Shifts

Sex differences in *P. lividus* gonads must be interpreted against a background of strong biological modulation by reproductive stage, seasonality, habitat, and diet, all of which can reshape gonadal resource allocation and biochemical composition [31,32,33]. This is directly relevant here because the polar metabolome integrates both (i) osmotic/energetic homeostasis of gonadal somatic tissue and (ii) biosynthetic demand from gametes, whose relative proportions may shift with sex and maturity. In this context, the betaine/TMAO axis aligns with a broader osmolyte strategy framework typical of marine invertebrates, while coordinated variation in free amino acids supports sex-linked differences in nitrogen/osmolyte budgeting rather than isolated single-metabolite effects [28,29]. Notably, sex-dependent chemical fingerprints in *P. lividus* gonads have also been recovered using complementary chemistry/chemometrics approaches, reinforcing that sex can be a detectable organizing factor even when other ecological drivers contribute substantial inter-individual variance [3,17].

A relevant point of comparison is our previous GC–MS study on sex-dependent aroma compounds in *Paracentrotus lividus* gonads, which likewise identified sex as a chemically meaningful source of variation in this tissue [17]. However, the two analytical platforms interrogate different layers of gonadal chemistry and should therefore be interpreted as complementary rather than directly equivalent. In the present work, ^1^H NMR primarily captures abundant polar metabolites associated with amino-acid and nitrogen metabolism, osmotic balance, energy-buffering pools, and nucleotide-related compounds, together with broad lipid motifs and unsaturation-related descriptors in the apolar fraction. By contrast, GC–MS without derivatization is inherently oriented toward volatile and aroma-active compounds, many of which may arise from low-abundance precursors, oxidation/degradation processes, or other secondary transformations. Accordingly, a direct one-to-one pathway reconstruction linking the two datasets would be overly speculative. The main convergence between both studies lies instead at the systems level: despite targeting different chemical spaces, both approaches consistently indicate that sex contributes to the organization of gonad chemistry in *P. lividus*. This complementary interpretation is more consistent with the analytical scope of each platform than a forced metabolite-by-metabolite correspondence. Future studies integrating NMR, targeted GC–MS, and species-resolved lipidomics on the same biologically staged specimens will be necessary to connect primary metabolite pools, lipid composition, and aroma-active volatiles within a more unified biochemical framework.

### 4.5. Interpretive Scope, Limitations, and Recommended Confirmatory Steps

Two limitations should be stated explicitly. First, because gonadal metabolite pools can vary strongly with reproductive stage and season in *P. lividus*, the absence of histological staging (or a robust proxy such as gonadosomatic index coupled to maturity scoring) limits mechanistic attribution of sex effects versus maturity effects and their interaction [31,32,33]. Second, some top discriminant resonances remain unassigned or tentative, so follow-up confirmation by targeted spiking and/or 2D NMR (HSQC/TOCSY-type support) would increase confidence in metabolite identity and downstream pathway interpretation. From a chemometrics perspective, given the near-perfect separation observed in score space, it is also recommended to document permutation testing and/or double cross-validation as an additional safeguard against overfitting, even when MCCV performance is excellent. Overall, the convergence of (i) full-spectrum PLS–LDA discrimination (Figure 2), (ii) SPA/COSS-prioritized biomarkers (Figure 3), and (iii) pathway-level representation (Figure 4) supports a robust sex-linked structure in the polar gonad metabolome dominated by amino-acid pools, osmolyte strategies, and biosynthetic precursor pathways—while also defining clear next steps for a higher-confidence mechanistic narrative.

Locality and habitat type were considered potential sources of variability in the present field design, particularly because spatial pseudoreplication can confound biological interpretation when all specimens are collected from a single cove or site. For this reason, sampling was distributed across two coastal sectors of Alicante Province under broadly similar shallow Mediterranean conditions and included both natural rocky habitats and artificial breakwater habitats. However, exploratory PCA did not reveal any clear or consistent clustering associated with either geographical origin or habitat category. Within the restricted spatial and temporal window considered here, sex therefore emerged as the dominant organizing factor in the metabolomic data. This does not exclude subtler locality- or habitat-related effects, but it indicates that such effects were not evident enough to justify stratified modelling in the present cohort.

### 4.6. Interpreting Apolar ^1^H NMR Lipid Signatures in Paracentrotus lividus Gonads: Ecological Drivers, Limitations, and Next Steps

Apolar ^1^H NMR features and biological interpretation: The apolar extracts of *Paracentrotus lividus* gonads displayed a lipid-dominated ^1^H NMR profile consistent with complex mixtures of acyl-chain signals (terminal –CH_3_, methylene envelope, allylic and bis-allylic –CH_2_–, and olefinic resonances) together with glycerol/ester-related features (Figure 5). While the dominant pattern is compatible with triacylglycerol-rich neutral lipids, the additional presence of resonances attributable to phospholipid-associated moieties suggests that membrane lipids contribute measurably to the apolar fraction and may vary, at least in part, independently from storage triacylglycerols. This interpretation is consistent with prior evidence that *P. lividus* gonads contain multiple phospholipid classes (including PI, PS, PE, PC and SM) and that, over the reproductive cycle, gonadal lipid extracts are typically TAG-dominated while phospholipids and DAGs occur at comparatively low abundance yet may still exhibit biologically meaningful dynamics [32,34,35].

Using the Bratu-type integral framework [24], the gonadal lipid composition was broadly similar between sexes for global indices of saturation/unsaturation and DHA. In the Bratu-derived subset (*n* = 34; 23 males, 11 females), three female samples were excluded because values fell outside the validity bounds of the Bratu model [24]. After exclusion, female and male medians remained close for unsaturated and saturated fractions (Mann–Whitney *p* ≈ 0.14 for both), and DHA %mol showed no statistically supported sex effect (*p* ≈ 0.36). In contrast, ω-3%mol exhibited a modest but significant difference (*p* ≈ 0.025), suggesting that any sex-associated shift—if present—may be more readily captured by an aggregate ω-3 descriptor than by a DHA-specific estimate. At the same time, it should be noted that (i) the post-filtering female sample size is smaller (*n* = 11), which limits power to detect subtle effects, and (ii) these NMR-derived indices intentionally compress compositional information into a small number of integrals and assumptions; therefore, they should be interpreted as robust, reproducible “coarse-grained” descriptors rather than a replacement for lipid-class or molecular-species resolution.

Ecological drivers likely dominate inter-individual lipid variability. In *P. lividus*, gonadal lipid pools are strongly shaped by reproductive stage and seasonal forcing, with documented annual dynamics in total lipids and fatty-acid profiles linked to temperature/photoperiod and maturation state [33,34,36]. Habitat and diet further modulate lipid availability and provisioning: field evidence shows contrasting lipid metabolism between habitats with different food sources, alongside selective storage of key LC-PUFAs (notably EPA and ARA) and their precursors, consistent with retention and/or bioconversion processes that are not trivially predicted by bulk diet composition [32]. Collectively, these mechanisms provide a coherent explanation for observing limited separability between sexes in Bratu-derived indices while still detecting meaningful inter-individual dispersion—particularly in ω-3-related metrics—within a mixed field dataset.

Scope, limitations, and recommended additions. The present apolar readout is informative but mechanistically underdetermined: (i) the dataset represents a cross-sectional snapshot and does not explicitly model gonadal stage (histology/GSI) or short-term diet history; (ii) ^1^H NMR lipid indices cannot resolve lipid molecular species (TAG/PL subclasses, positional distributions, oxidized lipids) and therefore cannot directly attribute ω-3 shifts to specific biochemical routes; and (iii) the Bratu model requires validity constraints, as reflected by the exclusion of out-of-range spectra [24]. To strengthen a high-confidence mechanistic narrative, the most impactful additions would be: targeted fatty-acid profiling (GC-FID/GC–MS) and/or LC–MS/MS lipidomics to resolve lipid classes and species; explicit staging of gonads (GSI + histology) to disentangle maturation effects; and, where feasible, controlled feeding/conditioning experiments to map diet-driven changes in gonadal PUFA pools and to test sex-by-diet interactions under defined inputs [37,38].

## 5. Conclusions

This study provides a reproducible ^1^H NMR-based framework to characterize both polar metabolites and apolar lipid motifs in *Paracentrotus lividus* gonads. For the polar fraction, we deliver a curated resonance inventory (chemical shifts and annotations) that captures the major biochemical classes expected in gonadal tissue—free amino acids and nitrogen carriers, compatible osmolytes/quaternary amines, energy-buffer metabolites, carbohydrates/aminosugars, and nucleotide-related compounds—thereby offering a practical reference for future sea urchin metabolomics, quality studies, and comparative surveys. Supervised chemometric modelling of polar fingerprints supports robust sex-linked differentiation, and SPA/COSS prioritization converges on coherent biochemical themes centred on amino-acid pools, osmolyte budgeting, and energy buffering, with pathway mapping reinforcing the prominent contribution of interconnected amino-acid and one-carbon/nucleotide networks.

In the apolar fraction, lipid-dominated spectra were consistent with mixtures of neutral lipids and membrane-associated glycerolipids, and equation-based lipid indices revealed high overall unsaturation and measurable ω-3 content but low DHA when benchmarked against fish oils, highlighting species- and tissue-specific lipid signatures relevant to nutritional context and valorisation perspectives. Overall, the combined polar–apolar profiling strategy, together with transparent reporting and curated annotation layers, establishes a solid baseline for subsequent work addressing seasonality, diet, habitat, reproductive stage, and processing-related effects on sea urchin gonad chemistry.

Future studies should integrate histological gonad staging, broader seasonal and environmental sampling, and complementary targeted lipidomic and volatilomic approaches to refine the biological interpretation of sex-related metabolic signatures in *Paracentrotus lividus* gonads.

## Figures and Tables

**Table 1:** Summary of the biological and biometric characteristics of the *Paracentrotus lividus* specimens included in the study.

Variable	Males	Females
Gonad samples analyzed, n	23	14
Test diameter (mm)	48.7 ± 6.5 (31.0–58.0)	52.5 ± 6.5 (41.0–65.0)
Test height (mm)	27.5 ± 3.7 (19.0–32.0)	29.6 ± 4.2 (24.0–38.0)
Total wet weight (g)	50.0 ± 15.4 (19.2–75.7)	54.5 ± 20.5 (25.0–100.1)
Gonad weight (g)	4.0 ± 2.3 (0.91–8.83)	3.7 ± 2.7 (0.90–11.91)
Gonadosomatic index (GSI, %)	7.4 ± 2.9 (3.92–12.87)	6.5 ± 2.9 (2.55–12.45)

**Table 2:** Complete annotated peak list for polar extracts: δH chemical shifts, multiplicities, proposed metabolite identities, and confidence/ambiguity flags for overlap-prone regions (aliphatic and aromatic).

Peak No.	δH (ppm)	Mult.	Metabolite	Moiety
1	0.9587	d	Valine	γ-CH_3_/γ′-CH_3_ (isopropyl methyls)
2	1.0000	s	Isoleucine (tentative)	δ-CH_3_ (terminal methyl)
3	1.0521	d	Valine	γ-CH_3_/γ′-CH_3_ (isopropyl methyls)
4	1.0955	—	Unassigned	—
5	1.3061	d	Threonine	γ-CH_3_
6	1.3390	d	Lactate	β-CH_3_
7	1.4315	m	Lysine (tentative)	Side-chain CH_2_ envelope (β/γ/δ overlap)
8	1.4715	d	Alanine	β-CH_3_
9	1.6708	m	Arginine (tentative)	β/γ-CH_2_ (side-chain methylenes)
10	1.7280	m	Lysine	δ-CH_2_ (side-chain methylene)
11	1.9052	s	Acetate	CH_3_
12	2.0630	s	N-acetylglucosamine (putative)	NAc-CH_3_ (acetamide methyl)
13	2.0816	m	Glutamate	β-CH_2_
14	2.1488	m	Glutamine	β-CH_2_
15	2.2327	s	Unassigned	—
16	2.3570	t	Glutamate	γ-CH_2_
17	2.4600	—	Glutamine (tentative)	γ-CH_2_
18	2.6797	s	Methylamine	CH_3_–NH_3_^+^ (methyl)
19	2.7913	s	Trimethylamine (tentative)	N(CH_3_)_3_
20	2.9100	m	Choline (tentative)/methylamine region	N-methyl region (overlap)
21	3.0362	t	Lysine	ε-CH_2_ (adjacent to NH_3_^+^)
22	3.1273	t	Histidine	β-CH_2_
23	3.2040	s	Choline/choline derivatives	N^+^(CH_3_)_3_ (trimethylammonium)
24	3.2298	s	Betaine	N^+^(CH_3_)_3_
25	3.2488	(s)	Trimethylamine oxide (TMAO)	N^+^(CH_3_)_3_ (amine oxide)
26	3.2665	t	Taurine	CH_2_–NH_3_^+^
27	3.3074	s	3,7-Dimethyluric acid (putative)	N-CH_3_ (xanthine/urate scaffold)
28	3.3539	s	Methanol	CH_3_OH
29	3.3813	—	Methanol (shoulder)/unassigned	—
30	3.410	s	Unassigned	
31	3.5042	m	Unassigned	CH_2_–O (carbohydrate/ribose/glycerol-type)
32	3.5101	m	Choline-related (probable)	CH_2_–O (choline moiety)
33	3.5648	s	Glycine	α-CH_2_
34	3.7611	t	Lysine	Hα (backbone CH)
35	3.7979	—	Alanine	Hα (backbone CH)
36	3.8532	m	Trehalose/carbohydrate (putative)	Sugar ring CH envelope
37	3.9000	—	Betaine (tentative)	CH_2_–COO−
38	3.9323	s	Creatine	CH_2_ (adjacent to guanidino system)
39	3.9800	—	Unassigned	—
40	4.3665	s	Trigonelline	N-CH_3_ (quaternary N-methyl)
41	5.7910	d/m	Uridine	H-5 (pyrimidine CH)
42	5.9940	d/m	Pyrimidine nucleoside (uridine/cytidine-like)	H-5 (pyrimidine CH)
43	6.1000	d	Purine riboside/ribonucleotide (inosine/IMP-like)	H-1′ (ribose anomeric proton)
44	6.8000	s	Phenolic aromatic (vanillic/vanillin-like, putative)	Aromatic ring CH
45	6.9070	d	Tyrosine	H-3,5 (AA′BB′ ring CH)
46	7.1260	s	Histidine	Imidazole CH (H-δ2)
47	7.1850	d	Tyrosine	H-2,6 (AA′BB′ ring CH)
48	7.2662	m	Phenylalanine	Aromatic ring CH envelope
49	7.2870	d/m	Tryptophan	Indole ring CH (H-5)
50	7.3250	m	Phenylalanine (tentative)	Aromatic ring CH envelope
51	7.3410	s	Imidazole	Ring CH (H-4/H-5)
52	7.4160	m	Phenylalanine	Aromatic ring CH envelope
53	7.5340	d/m	Tryptophan	Indole ring CH (H-7)
54	7.5500	S	Uracil	H-6 (pyrimidine CH)
55	7.6850	s	Unassigned	Aromatic CH (unknown)
56	7.7260	s/m	Xanthine and/or Tryptophan	Purine H-8 and/or indole aromatic CH
57	7.7730	s	Anserine (putative)	Imidazole CH (histidine-derived)
58	7.8510	s	N-acetyl-L-histidine (putative)	Imidazole CH
59	7.8620	d	Uridine	H-6 (pyrimidine CH)
60	7.882	s	Unassigned	Aromatic CH (unknown)
61	7.9730	t	Kynurenine-like (HSQC-consistent; downfield-shifted)	Aromatic ring CH
62	8.0000	s	Hypoxanthine	H-8 (purine CH)
63	8.0283	d	Kynurenine	--
64	8.0840	m	Trigonelline (reported)	Pyridinium ring CH
65	8.2351	s	Purine riboside/ribonucleotide (inosine/IMP-like)	Purine H-8 (ring CH)
66	8.2710	s	Imidazole	Ring CH (H-4/H-5)
67	8.3460	s	Purine riboside/ribonucleotide (inosine/IMP-like)	Purine H-2 (ring CH)
68	8.5454	t	Kynurenine	Pyridinium ring CH (overlapped)
69	8.5936	s	Adenine nucleotide (AMP-like)	Adenine H-8 (purine CH)
70	8.6998	m	Kynurenine	
71	8.8380	m	Trigonelline	Pyridinium ring CH (downfield member)
72	9.1260	s/m	Trigonelline	Pyridinium ring CH (most downfield)

**Table 3:** Apolar extract annotation layer: corrected δH positions and assignment of resonances to lipid moieties (TAG/DAG glycerol, fatty-acyl chain motifs, sterol/phospholipid-associated fragments), enabling downstream integral-based lipid indices.

Peak No.	δH (ppm)	Moiety	Main Lipid Class(es)	Proposed Assignment (Functional Group/Structural Element)
1	0.6803	Sterol methyl (C18–CH_3_)	Sterols (cholesterol-like)	Sterol “angular” methyl (18-CH_3_)
2	0.8806	Terminal –CH_3_	FA in TAG/DAG/MAG/PL	Terminal methyl of fatty acyl chains (major lipid methyl envelope)
3	0.9739	ω-3 terminal –CH_3_	ω-3 PUFA in TAG/PL	ω-3 methyl (EPA/DHA-type chains)
4	1.1150	Aliphatic methyl/methylene	Sterols/FA (minor)	Minor aliphatic resonance (sterol methyls/overlapping lipid methylenes)
5	1.2563	–(CH_2_)n–	FA in TAG/DAG/MAG/PL	Bulk methylene envelope of fatty acyl chains
6	1.3022	–(CH_2_)n–	FA in TAG/DAG/MAG/PL	Bulk methylene envelope of fatty acyl chains
7	1.4978	Aliphatic –CH_2_–	TAG/PL (overlap)	Mixed aliphatic region
8	1.6032	β-CH_2_ to C=O	TAG/PL (non-DHA enriched)	β-CH_2_ to carbonyl (acyl groups; “general” lipid region)
9	1.6757	β-CH_2_ to C=O	PUFA-enriched TAG/PL	β-CH_2_ to carbonyl (often increases with PUFA-rich acyl distributions)
10	1.8282	Aliphatic/allylic overlap	TAG/PL/sterols	Minor overlapped aliphatic signal
11	2.0202	Allylic –CH_2_–CH=CH–	UFA in TAG/PL	Allylic methylene (unsaturated acyl chains)
12	2.0780	Allylic –CH_2_–CH=CH–	UFA in TAG/PL	Allylic methylene (unsaturated acyl chains)
13	2.3079	α-CH_2_ to C=O	TAG/PL/FFA	α-CH_2_ to carbonyl (acyl groups; broad “F1-like” region)
14	2.3869	α-CH_2_ to C=O (DHA-sensitive window)	DHA-containing TAG/PL and/or DHA (FFA)	DHA marker window (α-CH_2_ near carbonyl for DHA acyl groups/DHA region)
15	2.4764	Minor	Trace/unknown	Unassigned weak resonance
16	2.7722	Bis-allylic –CH=CH–CH_2_–CH=CH–	PUFA (ω-6/ω-3)	Bis-allylic methylene (PUFA)
17	2.8094	Bis-allylic –CH=CH–CH_2_–CH=CH–	PUFA (ω-6/ω-3)	Bis-allylic methylene (PUFA)
18	2.8424	Bis-allylic –CH=CH–CH_2_–CH=CH–	PUFA (ω-6/ω-3)	Bis-allylic methylene (PUFA)
19	3.1409	Headgroup methylenes	PL (PE/others)	Putative ethanolamine-related methylenes (PE-type region)
20	3.3273	N^+^(CH_3_)_3_	PL (PC/SM)	Choline trimethylammonium (PC/SM “total choline” region)
21	3.4892	Residual solvent proton(s)	—	MeOH trace (residual solvent/contamination)
22	3.5343	Sterol H-3 (–CHOH)	Sterols	Sterol C3 proton (H-3, cholesterol-like)
23	3.7723	–CH_2_–O– (backbone)	DG/MG and/or PL	Glycerol/PL backbone region (overlap: DG/MG glycerol + PL backbone methylenes)
24	3.9490	–CH_2_–O–P– (phosphodiester-linked methylene)	PL	Phospholipid backbone region (CH_2_O–P/adjacent backbone methylenes)
25	4.1673	–CH_2_–O– (glycerol)	DG/MG and/or PL	Glycerol backbone methylenes (MG/DG) with possible PL overlap
26	4.2848	–CH_2_–O– (glycerol)	DG (1,2-DG) and/or PL	DG/PL backbone methylenes (often used for partial glycerides; overlaps PL)
27	4.3841	–CH_2_–CH_2_–N^+^(CH_3_)_3_ (choline methylenes)	PL (PC/SM)	Choline methylene region (PC/SM headgroup side-chain)
28	5.2784	sn-2 glycerol –CH–O–	TAG (± overlap)	TAG glycerol sn-2 CH
29	5.3654	Olefinic –CH=CH– (± sterol H-6 overlap)	UFA in TAG/PL + sterols	Olefinic protons of unsaturated acyl chains; can overlap sterol H-6

Docosahexaenoic acid (DHA), triacylglycerols (TAG), diacylglycerols (DAG), phospholipids (PL), free fatty acids (FFA), polyunsaturated fatty acids (PUFA)

## Data Availability

The data presented in this study is available on request from the corresponding author.

## Supplementary Materials

The following supporting information can be downloaded at: https://www.mdpi.com/article/10.3390/metabo16030211/s1. Table S1. MetaboAnalyst pathway analysis based on the complete set of polar metabolites detected in *Paracentrotus lividus* gonad extracts. The input compound list comprised all metabolites annotated from the ^1^H NMR spectra of the polar fraction across the full cohort (males and females combined), i.e., without restricting the analysis to sex-discriminatory features. The table reports, for each KEGG pathway, the total number of compounds in the pathway (Total), the expected number of hits under the null hypothesis (Expected), the number of matched metabolites from the input list (Hits), the nominal over-representation *p*-value (Raw p), the corresponding −log10(p), the Holm-adjusted *p*-value, the false discovery rate (FDR), and the pathway impact score from topology analysis (Impact). Pathways are sorted by increasing Raw *p*-value.
