# Supplementary material for: Sex-Driven Variation in Polar Metabolites and Lipid Motifs of Paracentrotus lividus Gonads Profiled by 1H NMR
Source: Metabolites. 2026 Mar 21;16(3):211. doi: 10.3390/metabo16030211 (PMC13028831; doi:10.3390/metabo16030211)
Supplement: Supplementary file 1 [file metabolites-16-00211-s001.zip › metabolites-4192450-supplementary.pdf]

Article

Sex-driven variation in polar metabolites and lipid motifs of *Paracentrotus lividus* gonads profiled by ^{1}H NMR

Ricardo Ibanco-Cañete [1], Estela Carbonell-Garzón [1], Sergi Amorós-Trujillo [2], Pablo Sanchez-Jerez [1] and Frutos C. Marhuenda Egea [2,*]

[1] Department of Marine Sciences and Applied Biology, University of Alicante, Carretera San Vicente del Raspeig s/n, 03690 Alicante, Spain; ricardo.ibanco@gmail.com (R.I.-C.); estela.carbonell@ua.es (E.C.-G.); pablo.sanchez@ua.es (P.S.-J.)

[2] Department of Biochemistry and Molecular Biology and Agricultural Chemistry and Edafology, University of Alicante, Carretera San Vicente del Raspeig s/n, 03690 Alicante, Spain

* Correspondence: frutos@ua.es

Academic Editor: Firstname Lastname

Received: date
Revised: date
Accepted: date
Published: date

Copyright: © 2026 by the authors. Submitted for possible open access publication under the terms and conditions of the Creative Commons Attribution (CC BY) license.

Table S1. MetaboAnalyst pathway analysis based on the complete set of polar metabolites detected in *Paracentrotus lividus* gonad extracts. The input compound list comprised all metabolites annotated from the ^{1}H NMR spectra of the polar fraction across the full cohort (males and females combined), i.e., without restricting the analysis to sex-discriminatory features. The table reports, for each KEGG pathway, the total number of compounds in the pathway (Total), the expected number of hits under the null hypothesis (Expected), the number of matched metabolites from the input list (Hits), the nominal over-representation p-value (Raw p), the corresponding –log10(p), the Holm-adjusted p-value, the false discovery rate (FDR), and the pathway impact score from topology analysis (Impact). Pathways are sorted by increasing Raw p-value.

Pathway	Total	Expected	Hits	Raw p	-log10(p)	Holm adjust	FDR	Impact
**Valine, leucine and isoleucine biosynthesis**	8	0.17992	3	0.00053	3.2758	0.040274	0.040274	0
**Phenylalanine, tyrosine and tryptophan biosynthesis**	4	0.08996	2	0.002848	2.5455	0.21358	0.058856	1
**Histidine metabolism**	14	0.31486	3	0.003145	2.5024	0.23273	0.058856	0.27083
**Purine metabolism**	71	1.5968	6	0.003852	2.4143	0.2812	0.058856	0.23668
**Arginine biosynthesis**	15	0.33735	3	0.003872	2.4121	0.2812	0.058856	0.25252
**Glycine, serine and threonine metabolism**	34	0.76466	4	0.005971	2.2239	0.42395	0.07516	0.07231
**Nitrogen metabolism**	6	0.13494	2	0.006923	2.1597	0.48458	0.07516	0
**Alanine, aspartate and glutamate metabolism**	23	0.51727	3	0.013355	1.8743	0.92151	0.12687	0.43991
**Phenylalanine metabolism**	9	0.20241	2	0.015933	1.7977	1	0.13454	0.44
**beta-Alanine metabolism**	15	0.33735	2	0.042758	1.369	1	0.30599	0
**Arginine and proline metabolism**	36	0.80964	3	0.044288	1.3537	1	0.30599	0.15356
**Pyrimidine metabolism**	41	0.92209	3	0.061359	1.2121	1	0.3886	0.11722
**Pantothenate and CoA biosynthesis**	20	0.4498	2	0.072219	1.1413	1	0.4222	0
**One carbon pool by folate**	26	0.58474	2	0.1138	0.9439	1	0.61777	0.08187
**Glyoxylate and dicarboxylate metabolism**	32	0.71968	2	0.1601	0.7956	1	0.79235	0
**Taurine and hypotaurine metabolism**	8	0.17992	1	0.16681	0.7778	1	0.79235	0.42857
**Tryptophan metabolism**	36	0.80964	2	0.1927	0.7151	1	0.81647	0.25849
**Caffeine metabolism**	10	0.2249	1	0.20412	0.6901	1	0.81647	0
**Biotin metabolism**	10	0.2249	1	0.20412	0.6901	1	0.81647	0
**Valine, leucine and isoleucine degradation**	40	0.8996	2	0.22615	0.6456	1	0.85937	0
**Butanoate metabolism**	15	0.33735	1	0.29047	0.5369	1	1	0
**Selenocompound metabolism**	18	0.40482	1	0.33787	0.4713	1	1	0

**Ubiquinone and other terpenoid-quinone biosynthesis**	21	0.47229	1	0.38221	0.4177	1	1	0
**Pyruvate metabolism**	23	0.51727	1	0.41016	0.387	1	1	0
**Glycolysis or Gluconeogenesis**	26	0.58474	1	0.44982	0.347	1	1	0
**Glutathione metabolism**	26	0.58474	1	0.44982	0.347	1	1	0.02912
**Tyrosine metabolism**	29	0.65221	1	0.4869	0.3126	1	1	0.12559
**Lysine degradation**	30	0.6747	1	0.49871	0.3021	1	1	0
**Porphyrin metabolism**	30	0.6747	1	0.49871	0.3021	1	1	0
**Glycerophospholipid metabolism**	36	0.80964	1	0.56428	0.2485	1	1	0.02771
**Amino sugar and nucleotide sugar metabolism**	43	0.96707	1	0.63035	0.2004	1	1	0.07692
